# Strategy of Residual Stress Determination on Selective Laser Melted Al Alloy Using XRD

**DOI:** 10.3390/ma13020451

**Published:** 2020-01-17

**Authors:** Yujiong Chen, Hua Sun, Zechen Li, Yi Wu, Yakai Xiao, Zhe Chen, Shengyi Zhong, Haowei Wang

**Affiliations:** 1 State Key Laboratory of Metal Matrix Composites, Shanghai Jiao Tong University, Shanghai 200240, China; yujiongchen@163.com (Y.C.); sunhua89@sjtu.edu.cn (H.S.); yakai.xiao@foxmail.com (Y.X.); hwwang@sjtu.edu.cn (H.W.); 2 SJTU-ParisTech Elite Institue of Technology, Shanghai Jiao Tong University, Shanghai 200240, China; 3 Beijing Institute of Astronautical Systems Engineering, Beijing 100076, China; 7823335@163.com; 4 School of Materials Science and Engineering, Shanghai Jiao Tong University, Shanghai 200240, China

**Keywords:** selective laser melting, residual stresses, X-ray diffraction (XRD), surface roughness, AlSi10Mg

## Abstract

Selective laser melting (SLM) is known to generate large and anisotropic residual stresses in the samples. Accurate measurement of residual stresses on SLM-produced samples is essential for understanding the residual stress build-up mechanism during SLM, while a dramatic fluctuation can be observed in the residual stress values reported in the literature. On the basis of studying the influence of surface roughness on residual stress measured using X-ray diffraction (XRD), we propose a procedure coupling XRD technique with pretreatment consisting of mechanical polishing and chemical etching. The results highlight that residual stresses measured using XRD on as-built SLM-produced samples with high surface roughness are significantly lower than those measured on samples with finished surface, which is due to the stress relaxation on the spiked surface of as-built samples. Surface distribution of residual stresses and the effect of scanning strategy were systematically investigated for SLM-produced AlSi10Mg samples. Microstructural morphology was observed at the interface between sample and building platform and was linked to the surface distribution of residual stresses. This procedure can help us accurately measure the residual stresses in SLM-produced samples and thus better understand its build-up mechanism during the SLM process.

## 1. Introduction

As one of the emerging additive manufacturing technologies, selective laser melting (SLM) has been widely studied in recent years. It shows a huge potential in producing high quality parts with complex geometry that would be difficult or expensive to produce with conventional subtractive manufacturing methods [1]. However, SLM is known to generate large and anisotropic residual stresses that can result in geometric distortion and severely affect mechanical performances [2]. Residual stresses in SLM-produced samples originate from steep temperature gradient (10^3^–10^4^ K/m [3]) in laser irradiated zone or from constrained shrinkage during rapid cooling (cooling rate 10^3^–10^8^ K/s [4]). Because of the layer-by-layer production process and the resulting complex thermal history, the build-up mechanism of residual stresses during SLM processes is not yet fully understood. P. Mercelis et al. [5] explained it by temperature gradient mechanism (TGM) and cool-down mechanism which are both based on the thermal expansion or shrinkage behavior of the material during rapid heating or cooling. Efforts have also been made to predict residual stresses and distribution by constructing analytical model [5] or using finite element method (FEM) [6].

Accurate experimental measurements of residual stresses in SLM-produced samples are vital not only for studying the residual stress build-up mechanism during SLM processes but also for calibrating FEM or analytical models. A number of experimental investigations of residual stresses in SLM-produced samples using different measurement methods have been reported. Destructive distortion-based methods (such as hole-drilling method [7] and contour method [6,8,9]) can estimate residual stresses in the sample by measuring its deformation when the samples with internal residual stresses are cut, but the accuracy of the estimation depends on the accuracy of the deformation measurement. Contrastingly, diffraction methods (such as X-ray diffraction (XRD) [10] and neutron diffraction (ND) [11,12]) are non-destructive. They can be used to obtain full-field strain/stress distribution by measuring at multiple positions on the sample surface [13,14,15,16] or can be combined with material removal techniques to acquire the in-depth distribution of residual stresses [17,18,19,20,21]. Nowadays, XRD becomes major non-destructive residual stresses measurement method because of its relative affordability and easy accessibility.

Although XRD is widely used to estimate the residual stresses, there is a debate on the obtained residual stress values and the measurement procedure for SLM-produced samples. Some studies revealed that the residual stresses were frequently shown to approach the material’s yield strength [19,22,23], while residual stresses reported for SLM-produced AlSi10Mg (summarized in Table 1) were far lower than its yield strength reported in the literature (200–300 MPa [24,25,26,27]). Moreover, a significant fluctuation was observed in the reported values and the sign of the surface residual stress cannot be determined properly. Up to now, there is no clear explanation for such a fluctuation. As most reported residual stress measurements using XRD were performed on as-built SLM-produced samples without considering the effect of surface roughness [14,22,28,29,30,31,32], some researchers consider the pretreatment prior to XRD measurements is necessary because the high surface roughness of the as-built samples can lead to inaccurate measurement of residual stresses [5,18]. For example, P. Mercelis et al. [5] proposed electrical discharge machining (EDM) and chemical etching before XRD measurements to obtain the actual residual stresses for SLM-produced 316L stainless steel samples. The purpose of chemical etching before performing XRD measurement was to remove the large tensile residual stress (~400 MPa) introduced during the EDM operation. In order to mitigate the effect of surface roughness on XRD measurements, S. Bagherifard et al. [18] applied electro-polishing to SLM-produced AlSi10Mg samples prior to XRD measurements.

Performing pretreatment using chemical etching or electro-polishing before XRD measurements seems able to obtain accurate residual stress values for SLM-produced samples. However, inappropriate treatment techniques may introduce extra residual stress and cause a redistribution of the original residual stress generated during SLM processes. An appropriate pretreatment technique is expected to reduce the surface roughness of the as-built SLM-produced samples without introducing additional residual stresses or altering the original residual stress distribution. Therefore, in this study, we propose a residual stress measurement procedure coupling XRD with pretreatment consisting of mechanical polishing and chemical etching to accurately measure the residual stresses on SLM-produced AlSi10Mg samples. The effect of sample surface roughness on residual stress measured using XRD was investigated. Surface distribution of residual stresses was given by multiple measuring points. The effect of scanning strategy on residual stress was investigated. The microstructural morphology was observed at the interface between sample and building platform and was linked to residual stress distribution. The proposed procedure can help us accurately measure the residual stresses in SLM-produced samples and thus better understand its build-up mechanism during the SLM process.

## 2. Experimental Procedures

### 2.1. Sample Preparation

**Figure 1:**
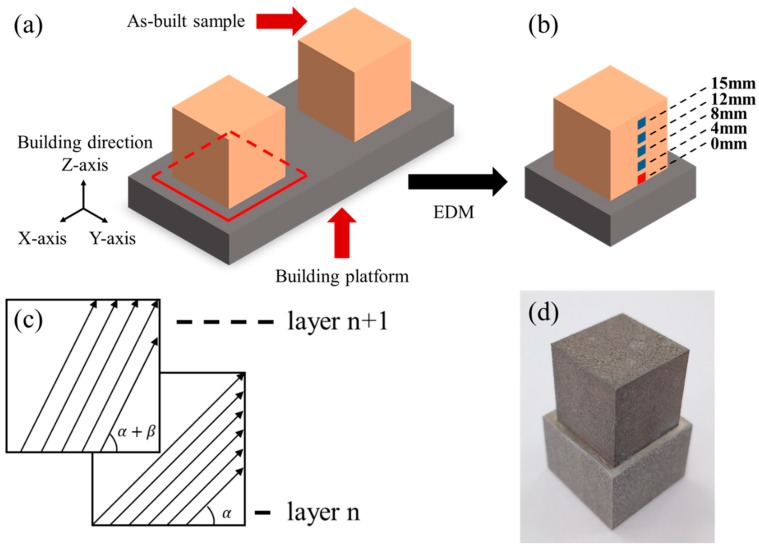
(**a**) Schematic representation of electrical discharge machining (EDM) processing on as-built samples. EDM cutting path are represented by red lines. (**b**) Schematic representation of XRD measuring points. Measurements at 0 mm (red area) on same sample before and after surface finishing were used to evaluate the effect of surface roughness on XRD measurements. Four additional measurements (blue area) were performed on surface finished sample to acquire residual stress distribution. (**c**) Representation of scanning strategies (starting angle α and rotation angle between subsequent layers β). (**d**) As-built sample cut together with building platform.

Commercial gas-atomized AlSi10Mg alloy powder supplied by VILORY Ltd, Jiangsu, China was used for SLM samples. The samples were manufactured using a ProX DMP 200 SLM machine (3D SYSTEMS, Wilsonville, OR, USA) that is characterized by a build chamber of 140 × 140 × 100 mm^3^, a maximum laser output of 300 W, a wavelength of 1070 nm, and a focused laser beam spot diameter of 75 μm. Based on previous studies, the process parameters were set to laser power of 200 W, scan speed of 1600 mm/s, layer thickness of 30 μm, and hatch spacing of 100 μm. The specimens were produced using five scanning strategies (Table 2) with different sets of starting angles (α) and rotation angles between subsequent layers (β), whose definition is shown in Figure 1c. The chamber was flooded with high purity argon to keep the oxygen content below 200 ppm. Cubic samples of 16 × 16 × 16 mm were considered for all experiments. In order to avoid releasing residual stresses when removing samples from the building platform, the specimens were kept attached to the platform and were cut together with the platform using electrical discharge machining (EDM) (Figure 1a,b).

### 2.2. Surface Finishing Procedures

In order to reduce surface roughness of as-built SLM-produced AlSi10Mg samples and avoid excessive removal of surface material, the samples were first ground manually using SiC paper (2500 grit) and polished using water-based diamond suspensions up to an average scratch size of 1.5 μm. About 150 μm-thick surface material was removed during mechanical grinding and polishing. The average particle diameter of P2500 grit SiC paper defined in ISO/FEPA Grit designation is 8.4 μm and was used to estimate the thickness of the surface material affected by mechanical grinding and polishing. A 50-μm-thick surface layer was removed by chemical etching as described below to remove the residual stresses introduced during mechanical grinding and polishing on the sample surface. The composition of the solution used in chemical etching is 930 mL H_2_O, 50 mL HNO_3_ (69.5 wt %), and 20 mL HF (50 wt %). The samples were immersed in the solution at 85 °C for 5 min. Then, the samples were rinsed in an ultrasonic bath using a solution of water and acetic acid to remove insoluble reaction products remained on the sample surfaces. In total, about 200-μm-thick surface material was removed.

### 2.3. Surface Roughness Measurement

The surface roughness of the samples was characterized using a Zeiss Smartproof confocal microscope (Zeiss, Oberkochen, Germany). Images were acquired on as-built, mechanical-polished, and chemical-etched samples. The quality of the surface was characterized by the parameters Sa, Sp, Sv, Sq, Ssk, and Sku. Sa represents the average of the absolute value of all peaks and valleys with respect to the median plane. Sp is the height of the highest peak and Sv is the absolute value of the depth of the largest pit. Sq represents the root mean square of ordinate values, equivalent to the standard deviation of heights. Ssk (skewness) values represent the degree of bias of the roughness shape. Sku (kurtosis) value is a measure of the sharpness of the roughness profile. Values of Ssk near to zero and of Sku near to three indicates a symmetrical height distribution.

### 2.4. Residual Stress Measurement

XRD was used to obtain residual stress distribution on the sample surface. PROTO iXRD-Portable X-ray diffractometer (PROTO, Oldcastle, Canada) with a Cr Kα source and sin^2^ψ method was used at a diffraction angle of 139° corresponding to 311-reflex of aluminum. To investigate the effect of surface roughness on residual stress measured using XRD, eight measurements were performed respectively at the same position (red area in Figure 1b) of the same sample before and after the surface treatment. 

After validating the effectiveness of the proposed residual stress measurement procedure, four additional measurements (blue area in Figure 1b) on the lateral surface were performed on surface-finished sample. Residual stress distribution on the lateral surface is given by five measuring points with a distance to the building platform ranging from 0 to 15 mm. Two components (σ_xx_ and σ_zz_) were measured at each position. Measurements were also conducted on surface-finished samples produced with five different scanning strategies.

### 2.5. Microstructure Characterization

Samples were prepared using the standard metallographic techniques and etched with Keller’s reagent (the aqueous solution of 2.5 vol % HNO_3_, 1.5 vol % HCl, 1 vol % HF) for 15 s. Scanning electron microscopy (SEM, TESCAN MAIA3, TESCAN, Brno, The Czech Republic) was used to examine the microstructural morphology at the joint between the SLM-produced samples and building platform.

## 3. Results

### 3.1. Surface Roughness

**Figure 2:**
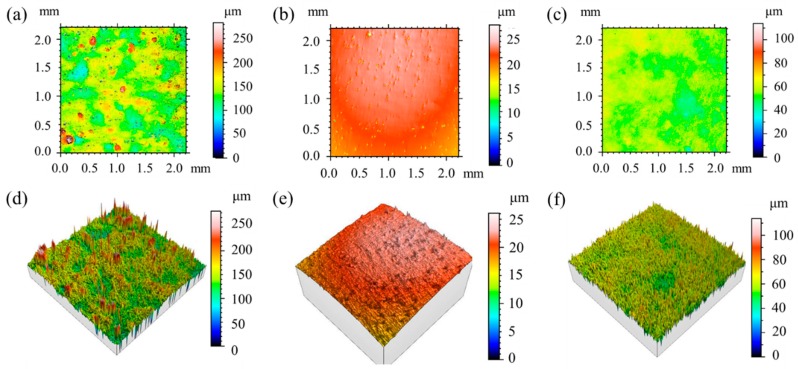
Surface morphology of (**a**,**d**) as-built; (**b**,**e**) mechanical-polished; (**c**,**f**) chemical-etched samples.

Surface morphology of as-built, mechanical-polished, and chemical-etched samples are shown in Figure 2. The parameters used to quantify the surface roughness are reported in Table 3, the values are an average of five measurements. As-built samples shows a high surface roughness with an average peak height Sa = 21.0 μm and a standard deviation Sq = 33.1 μm. Large values of Sp and Sv indicates the existence of high peaks and deep valleys compared to the median plane on the sample surface, as shown in Figure 2a,d. Moreover, large value of Sku indicates a highly spiked height distribution on as-built sample surface. Mechanical polished samples present a smooth surface (Figure 2b,e) with an average peak height Sa = 2.2 μm and a standard deviation Sq = 2.7 μm. After chemical etching (Figure 2c,f), Sa and Sq slightly increased to 6 μm and 7.8 μm respectively, but are still significantly lower than as-built samples. Sku of chemical machined sample slightly increased to 4.3, which indicates a slight spiked height distribution. It is worth noting that the chemical-etched samples have a Ssk value close to 0, which indicates the height distribution is symmetrical around the mean plane.

### 3.2. Residual Stresses

**Figure 3:**
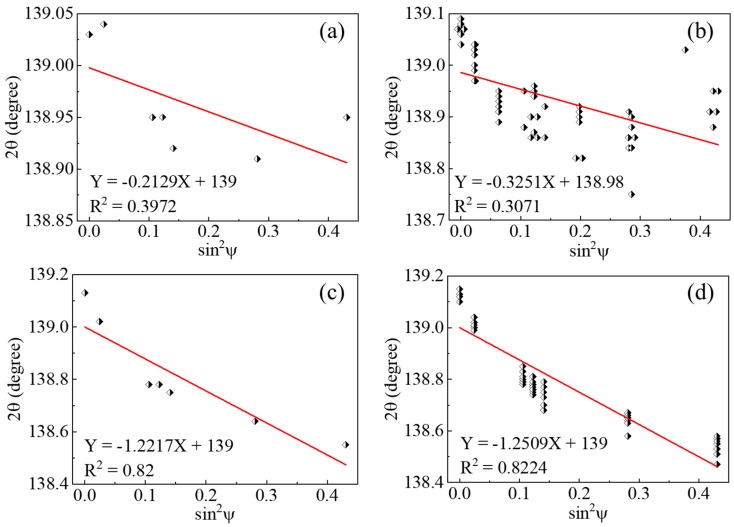
Plots of 2θ–sin^2^ψ for XRD measurements performed on as-built samples (**a**,**b**) and chemical-etched samples (**c**,**d**). Data points of single measurement was shown in (**a**,**c**) and a superposition of all data points obtained in eight measurements was shown in (**b**,**d**).

Taking 2θ and sin^2^ψ as ordinate and abscissa axis, the XRD data are plotted for measurements performed on as-built samples (Figure 3a,b) and chemical-etched samples (Figure 3c,d). Data points of single measurement are shown in Figure 3a,c while a superposition of all data points obtained in eight measurements is shown in Figure 3b,d. The slope of 2θ-sin^2^ψ straight line is obtained by linear fitting using ORIGIN standard software. The fitted relationship and corresponding coefficient of determination R^2^ are also plotted. Statistics of values obtained from XRD measurements are shown in Table 4, including the slope and the coefficient of determination (R^2^) of the fitted 2θ-sin^2^ψ line as well as the resulting residual stress. The average and the standard deviation were calculated based on results of eight measurements.

Linear fitting of XRD data acquired on chemical etched samples possesses a significantly larger coefficient of determination R^2^ compared to the as-built samples, which indicates the fitted line for chemical etched samples approximates much better the real data points. For chemical etched samples, the slope and R^2^ value obtained from linear fitting of the superposition of eight measurements (Figure 3d) gave similar results to the single measurement (Figure 3c) as well as the average reported in Table 4. In contrast, the data points of XRD measurements performed on as-built samples are highly discrete, characterized by a low value of R^2^ in Figure 3b, which indicates no clear linear relationship can be found. A significant fluctuation characterized by large coefficient of variation was observed for XRD measurements performed on as-built samples. It is worth noting that the coefficient of variation of residual stress is significantly smaller compared to the other two parameters.

**Figure 4:**
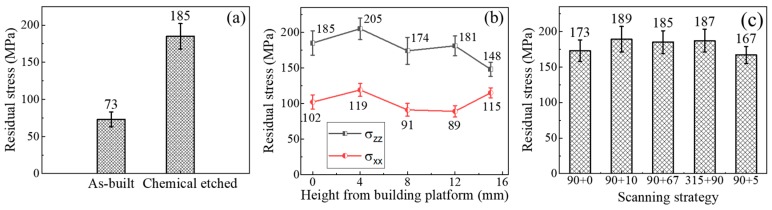
Residual stresses measured using XRD (**a**) at the same position of the same sample before and after surface treatment; (**b**) on lateral surface of the chemical etched sample; (**c**) on samples fabricated with five different scan strategies. Positive values represent tensile residual stresses.

Figure 4a shows the residual stress measured using XRD before and after surface treatment at same position (red area in Figure 1b) of the same sample. Value measured on chemical-etched sample (185 ± 16 MPa) is about 2.5 times larger than that measured on as-built sample (73 ± 10 MPa). Figure 4b shows the residual stress distribution on lateral surface of the chemical etched sample. σ_zz_ increased in magnitude to a peak value of 205 ± 15 MPa at 4 mm from the building platform and then decreased until reaching a minimum of 148 ± 9 MPa at 15 mm. σ_xx_ showed a similar tendency with a maximum of 119 ± 9 MPa and a minimum of 89 ± 8 MPa, except a sharp increase in magnitude at 15 mm. Residual stresses measured on samples produced with five different scanning strategies are summarized in Figure 4c. The measured values of σ_zz_ presented a rather constant tensile stress with an average of about 180 MPa.

### 3.3. Microstructure

**Figure 5:**
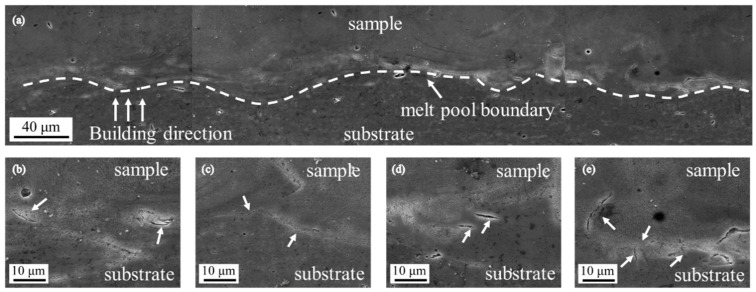
(**a**) SEM observation at the joint between SLM-produced AlSi10Mg samples and building platform. White dashed lines represent melt pool boundaries. (**b**–**e**) Higher magnification of cracks. White arrows point to cracks.

Figure 5 shows SEM images acquired on lateral surface of SLM-produced AlSi10Mg sample. Microstructural morphology at the joint between sample and building platform was investigated. Clear melt pool boundaries were indicated by white dashed lines in Figure 5a. Numerous cracks were observed near the joint and most of them are distributed on the sample side. The majority of observed cracks is propagated perpendicular to the building direction. A few cracks extending along the building direction were observed to cross the melt pool boundaries. Higher magnification of cracks was given in Figure 5b–e.

## 4. Discussion

### 4.1. Necessity of Surface Treatment before XRD Measurements

The big gap between residual stresses measured at same position before and after surface treatment shows that surface roughness can significantly affect residual stresses measured using XRD. Actually, the residual stress values given by XRD is the arithmetic average stress in a volume of material in the irradiated area. The intensity of the X-ray penetrating to a depth x can be described as [33]:

I(x) = I_0_ × e^(−μx)^ (1)

where I_0_ is the initial intensity of the X-ray, μ is the linear absorption coefficient, and e is the natural logarithm base (2.71828…). The linear absorption coefficient μ of aluminum-base alloys under Cr Kα radiation and at a diffraction angle of 139° is reported to be 44.3 mm^−1^ [33]. The half value depth, defined as the depth where the intensity of the X-ray is a half of the initial intensity, can be calculated to be about 16 μm for aluminum-base alloys. The calculated value is comparable to the X-ray penetration depth reported in the literature (~20 μm [5,34,35]).

If the surface roughness of the sample is comparable to the penetration depth, stress relaxation due to spiked sample surface can lead to lower residual stress measured using XRD. As shown in Table 3, average peak height (Sa) measured on as-built SLM-produced samples is about 21 μm and is comparable to penetration depth of X-ray in the material. Therefore, the significantly lower residual stresses measured on as-built SLM-produced samples is caused by its high surface roughness. In order to accurately measure the residual stresses on SLM-produced samples, surface treatment must be performed before XRD measurements to reduce the surface roughness of the samples.

### 4.2. Effectiveness of the Proposed Residual Stress Measurement Procedure for SLM-Produced AlSi10Mg Samples

The surface treatment method proposed in the present study effectively reduced surface roughness of the as-built samples and made the height distribution more symmetrical by removing a 200 μm-thick layer of surface material. The final average peak height (Sa) of chemical etched sample is 6.0 μm and is negligible compare to penetration depth of X-ray in the material. The total removed thickness of about 200 μm is negligible compare to the total height of the as-built samples (16 mm), the measured residual stresses can thus be considered as the surface residual stresses on SLM-produced samples. Multiple measurements performed at different positions on the surface finished samples gave different values, which can confirm the effective removal of the introduced residual stresses during mechanical grinding and polishing. 

As shown in Figure 3, the data points of XRD measurements carried-out on as-built samples are highly discrete. The resulting low R^2^ value and large coefficient of variation of the slope indicate that the residual stress measurements using XRD performed on as-builts samples are inaccurate and non-repeatable. Moreover, the residual stress measured on as-built samples fluctuate much less than the slope of the fitted line. Since the slope is used for calculating residual stresses in sin^2^ψ method, the observed inconsistency between the fluctuation of the slope and the resulting residual stress values further confirms that residual stresses measured using XRD on as-built samples are biased. 

Contrary to as-built samples, the much lower coefficient of variation for XRD measurements conducted on chemical etched samples indicates the stability and the good repeatability of the results. The significantly larger R^2^ values ensure the accuracy of the linear fitting and thus the resulting residual stresses. Therefore, it can be concluded that the proposed procedure can improve the accuracy and repetitiveness of the residual stress measurements for SLM-produced AlSi10Mg samples.

### 4.3. Residual Stress Distribution in SLM-Produced AlSi10Mg Samples

The residual stress profile in Figure 4b shows that residual stresses are unevenly distributed on the sample surface and measured values depend on measuring position. At each measuring point, σ_zz_ is 1.5 to 2 times larger than σ_xx_, which indicates the major component of residual stresses on the lateral surface of SLM-produced samples is along the building direction. 

The peak value of σ_zz_ occurred at 4 mm from the building platform but not at the interface between sample and building platform. This shift of the peak value can be attributed to low yield strength and ultimate tensile strength of AlSi10Mg alloy at high temperature. During SLM processes, residual stresses cumulated in the sample is released by plastic deformation or crack once exceeded the yield strength or ultimate tensile strength of AlSi10Mg alloy at high temperature. Cracks observed in Figure 5 are mainly distributed near the interface between sample and building platform, which indicates the existence of residual stresses superior to ultimate tensile strength of AlSi10Mg near the interface. Residual stresses formed during SLM processes at the interface were partially released by cracks during the fabrication processes. Moreover, most observed cracks propagate along a direction perpendicular to the building direction, which corresponds to σ_zz_ much larger than σ_xx_ on the lateral surface. It is also worth noting that the maximum of σ_zz_ (205 ± 15 MPa) is comparable to the yield strength of AlSi10Mg alloy at room temperature, which reveals that yield strength at room temperature limits the cumulated residual stresses in the sample when it is not released by cracks.

σ_zz_ measured on the lateral surface of the samples fabricated with five different scanning strategies shows a rather constant value with an average of 180 MPa. Actually, the only difference between five used scanning strategies is the scan pattern in the building plane, defined by starting angle and rotation angle. Since the difference is solely in the building plane and thus purely planar, it has little effect on residual stresses cumulated on the lateral surface.

## 5. Conclusions

In the present study, a residual stress measurement procedure for SLM-produced AlSi10Mg samples was developed by combining XRD with pretreatment including mechanical polishing and chemical etching. The key findings are summarized as follows:

- The proposed procedure can improve the accuracy and repetitiveness of the residual stress measurements for SLM-produced AlSi10Mg samples by reducing the effect of surface roughness on XRD measurements. This strategy of residual stress determination is also applicable for SLM-produced samples using other materials, especially for those with high surface roughness.
- High surface roughness of the as-built SLM-produced samples can lead to lower residual stress measured by XRD because of stress relaxation on the spiked surface. Because of its comparable surface peak height to X-ray penetration depth in the material, as-built samples need to be finished prior to XRD measurements.
- Residual stresses generated during SLM processes is unevenly distributed on sample surfaces. Residual stresses along the building direction (σ_zz_) is much larger than the component perpendicular to building direction (σ_xx_) on the lateral surface.
- Residual stresses generated during SLM processes at the interface between sample and building platform were partially released by cracks occurred during fabrication processes. For other positions where residual stresses are not released by cracks, the maximum value approaches to the yield strength of the material at room temperature.

## Figures and Tables

**Table 1:** Summary of residual stress measurements performed on selective laser melting (SLM)-produced AlSi10Mg samples reported in the literature.

Preheating Temperature (°C)	Post Processing	Measured Values (MPa)	Measurement Method	Measuring Surface	Reference
/	/	−75	XRD XRD	top lateral	[21] [28]
/	vibratory polishing	−130 to −90			
80	/	40	XRD	top	[31]
120	/	25			
160	/	10			
150	/	50 ± 30	XRD	lateral	[18]
	T6	−10 ± 10			
200	/	7.7 ± 5 to −6.4 ± 5	XRD	top	[29]
	200 °C/1 h	30.7 to 64.9		lateral	
	300 °C /2 h	0 to 20			
	T6	26.8 to 78.9			

**Table 2:** Five scanning strategies used in SLM fabrication defined by the starting angle α and the interlayer rotation angle β.

Scan Strategy	Starting Angle (α/°)	Rotation Angle between Subsequent Layers (β/°)
90 + 0	90	0
90 + 5	90	5
90 + 10	90	10
90 + 67	90	67
315 + 90	315	90

**Table 3:** Surface roughness parameters of as-built (AB), mechanical-polished (MP), and chemical-etched (CE) samples.

	Sa (μm)	Sp (μm)	Sv (μm)	Sq (μm)	Ssk	Sku
AB	21.0 ± 2.5	195.1 ± 33.2	147.9 ± 19.8	33.1 ± 4.4	0.871 ± 1.6	12.62 ± 3.3
MP	2.2 ± 0.1	8.1 ± 0.4	16.4 ± 2.4	2.7 ± 0.1	−0.8 ± 0.01	3.1 ± 0.01
CE	6.0 ± 0.2	57.3 ± 5.1	56.5 ± 4.2	7.8 ± 0.2	−0.05 ± 0.01	4.3 ± 0.1

**Table 4:** Statistics of data obtained from XRD measurements: slope and coefficient of determination (R^2^) of the fitted 2θ-sin^2^ψ straight line as well as the resulting residual stress (RS). Coefficient of variation (CV) is defined as the ratio of the standard deviation to absolute value of the average.

	As-Built			Chemical Etched		
	Slope	R^2^	RS (MPa)	Slope	R^2^	RS (MPa)
Average	−0.482	0.561	73.1	−1.251	0.837	185.2
Standard deviation	0.235	0.150	7.9	0.108	0.033	8.1
CV (%)	48.79	26.75	10.90	8.65	3.89	4.39
